# Soil drought sets site specific limits to stem radial growth and sap flow of Douglas-fir across Germany

**DOI:** 10.3389/fpls.2024.1401833

**Published:** 2024-08-06

**Authors:** Armin Niessner, Stefan Ehekircher, Reiner Zimmermann, Viviana Horna, Daniel Reichle, Alexander Land, Göran Spangenberg, Sebastian Hein

**Affiliations:** ^1^ Department of Silviculture, University of Applied Forest Sciences, Rottenburg am Neckar, Germany; ^2^ Ecological Botanical Gardens ÖBG, University of Bayreuth, Bayreuth, Germany; ^3^ Department of Biogeochemical Processes, Max Planck Institute for Biogeochemistry, Jena, Germany; ^4^ Institute of Biology, University of Hohenheim, Stuttgart, Germany

**Keywords:** *Pseudotsuga menziesii*, drought stress, non-native tree species, forestry, dendrometer, tree water deficit, climate change, water relations

## Abstract

**Introduction:**

Soil drought during summer in Central Europe has become more frequent and severe over the last decades. European forests are suffering increasing damage, particularly Norway spruce. Douglas-fir (*Pseudotsuga menziesii* (Mirbel) Franco), a non-native tree species, is considered as a promising alternative to build drought-resilient forests. The main goal of this study was to investigate the intraannual radial stem growth and sap flow performance of Douglas-fir along a precipitation gradient across Germany under severe drought.

**Material and methods:**

Sap flow and stem radial changes of up to ten trees each at four sites with different precipitation regimes were measured in combination with volumetric soil water content during the growing season of 2022. Measurements of stem radial changes were used to calculate the trees’ stem water deficit, a proxy for tree water status and drought stress.

**Results:**

The severe summer drought of 2022 led to an early growth cessation and a significant reduction in daily sap flow at all four sites monitored. We could identify a site-specific threshold in soil water availability ranging between 21.7 and 29.6% of relative extractable water (REW) under which stem water reserves cannot be replenished and thereby inhibiting radial growth. We could also demonstrate that at this threshold, sap flow is heavily reduced to between 43.5 and 53.3%, and for a REW below 50%, sap flow linearly decreases by 1.1–2.0% per 1% reduction in REW. This reduction tends to follow the humidity gradient, being more pronounced at the most oceanic characterized site and suggesting an adaptation to site conditions. Even though Douglas-fir is considered to be more drought stress resistant than Norway spruce, growth and sap flow are greatly reduced by severe summer drought, which became more frequent in recent years and their frequency and intensity is likely to increase.

**Conclusions:**

Our results suggest that timber production of Douglas-fir in Central Europe will decline considerably under projected climate change, and thus pointing to site specific growth constraints for a so far promising non-native tree species in Europe.

## 1 Introduction

Soil drought has become a dominant feature during summer over the last decades in Central Europe (Briffa et al., 2009; Petrow and Merz, 2009; Ruosteenoja et al., 2018; Intergovernmental Panel On Climate Change, 2023) and most studies forecast even higher temperatures and further shifts in precipitation patterns (Christensen and Christensen, 2004; Christensen et al., 2015; Huang et al., 2015). The occurrence of soil drought in Germany, documented through the German drought monitor (Zink et al., 2016), has raised significant concerns regarding the future of temperate tree species (broad leaved and conifer trees). The decreasing availability of water resources, as e.g., demonstrated by Köcher et al. (2009), exerts adverse effects on the hydric condition of these trees, leading to reduced growth (Ma et al., 2012; Land et al., 2017), reduced sap flow (Clausnitzer et al., 2011; Brinkmann et al., 2016) and increased mortality (Wang et al., 2012; Allen et al., 2015; Gessler et al., 2017). Of particular concern is the susceptibility of Europe’s main timber species (Caudullo et al., 2016), Norway spruce (*Picea abies* (L.) H. Karst.), to the threads of drought and bark beetle infestations (Dobbertin et al., 2007; Marini et al., 2017). Drought-related stress impairs tree growth and increases susceptibility to pathogens (Ma et al., 2012).

This has led to a reconsideration of the role and potential of non-native species in German forestry. Douglas-fir emerged as a focal point of interest for its ability to withstand drought stress. Originating from North America and introduced approximately 150 years ago, Douglas-fir (*Pseudotsuga menziesii* (Mirbel) Franco) has exhibited remarkable drought resilience compared to species native to Central Europe (Eilmann and Rigling, 2012; Nadezhdina et al., 2014; Vitali et al., 2017) and has now become one of the economically most significant non-native tree species in European forests (Kohnle, 2007; Brus et al., 2019). Its rapid growth and prolific biomass production, underscored by Dietz and Bürgi (1991), Hessemöller et al. (2001) and Miller et al. (2022), along with its favorable wood properties (Zeidler et al., 2017), even outperforming Norway spruce and Scots pine (Zeidler et al., 2022), and resistance to native fungal pathogens (Möller and Heydeck, 2009), have made it a crucial component of many of today’s forestry plans. Douglas-fir covered approximately 3% of the total potential forest area in France (ca. 420,000 ha) and 2% in Germany (ca. 220,000 ha), as reported by Kownatzki (2011) and Spiecker et al. (2019), respectively.

Continuous monitoring of stem radial changes using automated dendrometers can complement the conventional assessment of tree water status, measured through leaf water potential (pre-dawn and midday) using a Scholander pressure bomb (Scholander et al., 1965). These radial changes primarily result from two physiological processes: irreversible stem expansion due to growth and reversible stem size variations driven by hydration and dehydration, especially in the bark. The negative deviation from the previous maximum stem expansion is referred to as tree water deficit (TWD, Zweifel, 2016), which is markedly influenced by the tree’s water status and provides continuous, detailed assessments throughout entire seasons in mature trees and unveiling drought stress (Zweifel, 2016). The “zero growth concept” postulates that radial stem growth is only possible when the cells of the cambium are turgescent (Lockhart, 1965; Steppe et al., 2006), i.e. a prolonged TWD inhibits stem radial growth (Zweifel et al., 2016).

Furthermore, sap flow measurements are crucial for understanding the dynamics of tree water-use under drought conditions. If soil water is scarce, while evaporative demand of the atmosphere is high, trees will close their stomata to avoid dehydration of cells. This, however, strongly reduces carbon uptake. Low soil water potentials during soil drought induces critical xylem water potentials, which can cause cavitation and xylem dysfunction (Tyree and Sperry, 1989). As a result, xylem resistance to water flow is increased and maximum sap flow capacity is reduced (Willson and Jackson, 2006). E.g. Brinkmann et al. (2016) used such relations and showed for temperate angiosperm tree species a uniform threshold in soil moisture that induces TWD and a species-specific decline in sap flow.

In particular, the severe soil droughts that frequently occurred in Germany since 2018, as recorded by the German drought monitor (Zink et al., 2016), raise concerns about the future suitability of Douglas-fir to replace Norway spruce for high timber production (Podrázskỳ et al., 2016). To better understand whether Douglas-fir is indeed a suitable alternative in Central European forestry under dry conditions and under predicted future climate change, we studied the intraannual stem radial changes and xylem sap flow of ten Douglas-fir trees at four locations in Germany. Studies on drought response of Douglas-Fir in Central Europe are still scarce, so the primary goal of this study was to analyze the effects of soil drying during the summer drought of 2022 on Douglas-fir trees along a precipitation gradient across Germany. We hypothesized that:

1. Soil drying below a certain threshold prevents stem radial growth of Douglas-fir, leading to early growth cessation as a consequence of a persistent water deficit in the stem (TWD) analog to a threshold reported for angiosperm tree species by Brinkmann et al. (2016).
2. This threshold value for soil moisture and the response of sap flow to soil drying of Douglas-fir is site-specific and a consequence of adaptation to the growing conditions at the site.

## 2 Materials and methods

### 2.1 Study sites

**Figure 1:**
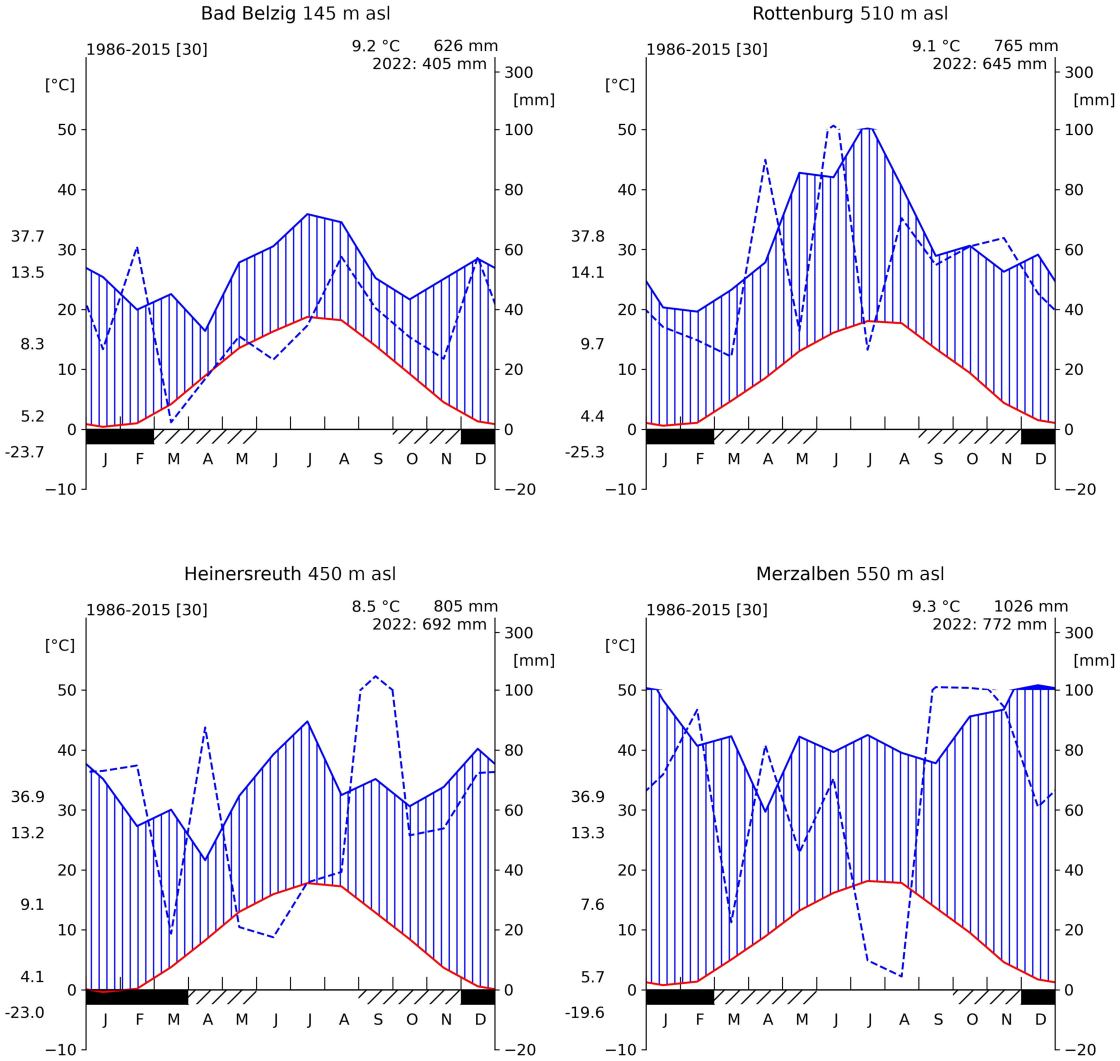
Climate diagrams of all four sites (1986–2015). For each panel, red line indicates the mean monthly temperature, blue solid line the mean monthly precipitation and blue dashed line shows monthly precipitation for the year 2022. Mean annual temperature (T) and mean annual sum of rainfall is shown at the top right corner. Along the left axis, from top to bottom, the maximum recorded T, the mean daily maximum T, the mean daily range in T, the mean daily minimum and the minimum recorded T. Solid black bar along the bottom axis indicates months with frost and hatched bar months with possible frost. Source data obtained from the DWD/BfG-HYRAS v2.0 data set (Rauthe et al., 2013; Brienen et al., 2016).

Four sites were selected along a gradient in annual precipitation and with different rainfall regimes throughout the year from east to west Germany. The driest and most continental site (BB) is located near Bad Belzig, Brandenburg, in the Hoher Fläming at 145 m a.s.l. While there is an average annual precipitation of 626 mm and a mean temperature of 9.2°C, most rainfall is received during summer months (**Figure 1**). Selected trees grow in an 80 years old pure Douglas-fir forest with abundant natural regeneration and with an average height of 34 m. These are the tallest trees compared to the following sites. A soil profile was taken in approximately 3 m distance to neighboring trees and with no other tree species nearby. We counted the coarse (diameter*>*2 *mm*) and fine roots (diameter*<*2 *mm*) along the soil profile using a metrical frame for tree-root measurements. Around 70% of the fine and coarse roots are found within the upper 25 cm of a silty sand soil and 90% of the roots are found within the upper 35 cm (**Table 1**; **Supplementary Figure S1**).

**Table 1:** Summary of site characteristics based on observations taken within one circular plot of radius 15 m (BB), 20 m (RO, HE) and 25 m (ME) containing all studied trees.

site	Bad Belzig (BB)	Rottenburg (RO)	Heinersreuth (HE)	Merzalben (ME)
Coordinates	52.166838° N 12.514308° E	48.446685° N 8.968711° E	49.960089° N 11.464497° E	49.273530° N 7.807540° E
Elevation [m a.s.l.]	145	510	450	550
Site age [years]	80	45	110	55
Average tree height [m]	34.0	29.3	31.5	32.5
Forest Site Index (m height at age 100)	37 (Site class II.25)	43 (Site class 0.75)	30 (Site class IV.0)	42 (Site class I.0)
Vegetation	Pure Douglas-fir (100% of BA) forest with abundant natural regeneration	Almost pure Douglas-fir (74% of BA) forest	Mainly Douglas-fir (88% of BA) forest with few European beech and oak trees	Mainly Douglas-fir (98% of BA) forest with few European beech trees
Stems ha^−1^ (*>*15 cm circumference)	668	560	1744	366
Basal area, BA [m² ha−1]	45.48	33.12	54.54	57.44
Soil texture	0–60cm: silty sand, 60–90 cm: slightly silty sand, below 90 cm: sand	0–45 cm: sandy silt, 65–45 cm: loamy clay, below 65 cm: slightly silty clay	0–45 cm: silty sand, below 45 cm: loamy clay	sandy silt
% of FR (CR) found up to 25 cm depth	71 (68)	69 (70)	49 (69)	38 (41)
max. depth of 90% of FR (CR) [cm]	35 (35)	40 (40)	45 (45)	55 (60)
max. VSWC [%]	22.3	21.2	34.5	27.2
min. VSWC [%]	6.4	6.2	14.9	5.8

Forest Site Index according to Noack (2021). FR, fine roots (diameter<2mm); CR, coarse roots (diameter>2mm). Maximum and minimum volumetric soil water content (max. and min. VSWC), corresponding to estimates for field capacity and permanent wilting point of soils, respectively. See also **Supplementary Figure S1** for soil texture and distribution of roots and **Supplementary Tables S1–S4** for individual tree characteristics.

The second site (RO) is in the Rammert Forest near Rottenburg, Baden-Württemberg. It lies at 510 m a.s.l. with an annual average precipitation of 765 mm and an annual mean temperature of 9.1°C. Rainfall is dominantly received during summer months (**Figure 1**). Trees were selected within a 45 years old almost pure Douglas-fir forest, however, already reaching heights of almost 30 m on average. This site is relatively open with only 560 stems and a basal area of 33 m² per hectare. Similar to BB, about 70% of the roots are found within the upper 25 cm of a sandy silt soil and 90% within the upper 40 cm. Between 45 and 65 cm, there is a layer of loamy clay (**Table 1**; **Supplementary Figure S1**).

A third site (HE) is located in the Heinersreuth Forest near Bayreuth, Bavaria at 450 m a.s.l. While the average annual precipitation is 805 mm and the annual mean temperature 8.5°C, rainfall is more evenly distributed throughout the year, but most rain is still received during summer (**Figure 1**). Trees were selected within a 110 years old-growth mixed Douglas-fir forest, reaching heights of 31.5 m on average. Besides Douglas-fir, also European beech (*Fagus sylvatica* L.), oak (*Quercus* sp.) and fir (*Abies alba* Mill.) occur, however, rather sparsely distributed. With 1744 stems and a basal area of 54.54 m² per hectare, this site is relatively dense. Trees predominantly root in a silty sand soil layer of 45 cm depth, whereas about 50% are found within the upper 25 cm (**Table 1**; **Supplementary Figure S1**).

The most oceanic site (ME) is located in the Palatinate Forest near Merzalben, Rhineland-Palatinate at 550 m a.s.l. With an annual average temperature of 9.3°C and 1026 mm of precipitation, it is the warmest and wettest of the four sites. Rainfall is evenly distributed throughout the year, but most rain is received during winter months (**Figure 1**). Trees were selected within a relatively young (55 years) Douglas-fir dominated forest, intermingled with few European beech trees. Tree heights reach 32.5 m on average and are sparsely distributed with only 366 stems per hectare, but a relatively high basal area with 57.44 m² per hectare. The upper 100 cm of the soil is continuously sandy silt, where only about 40% of roots are found within the upper 25 cm and 90% of roots are found up to a depth of 55 to 60 cm (**Table 1**; **Supplementary Figure S1**).

Soil types for all sites were determined using the test method of Boden (2005).

### 2.2 Meteorological data

**Figure 2:**
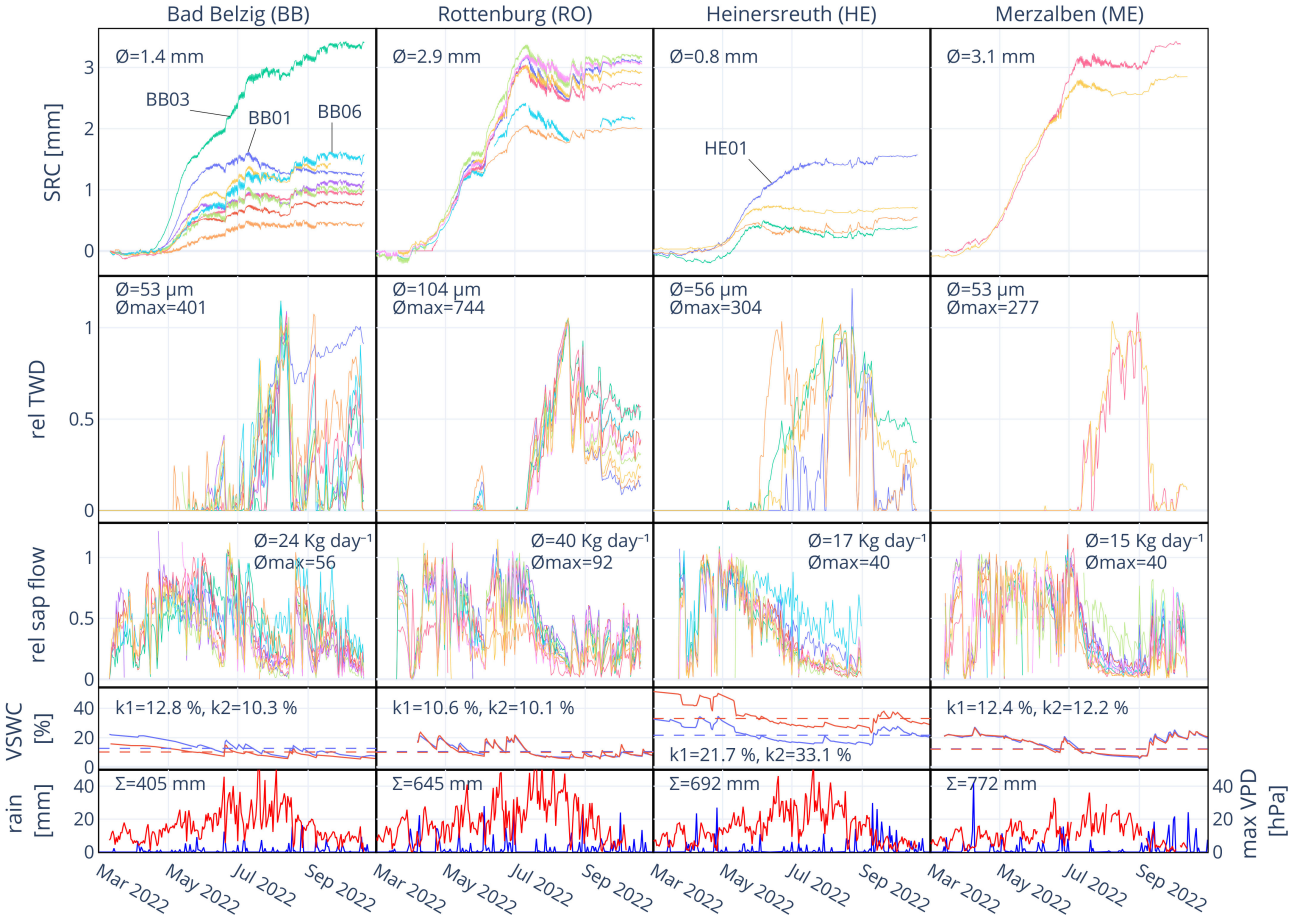
Stem radial change (SRC), relative tree water deficit (rel TWD), relative sap flow (rel sap flow), volumetric soil water content (VSWC) and rainfall at all sites from March to September 2022. Mean absolute SRC, mean daily TWD, mean maximum TWD, mean daily sap flow and mean maximum sap flow are indicated in the respective panels. k1 and k2 are the determined thresholds for VSWC in % for 20 (blue line) and 40 cm (red line) soil depth (60 cm in BB), respectively. The daily sum of rain (blue) and daily maximum VPD (red) during the time of observation are indicated in the bottom panels.

Data on long-term daily temperature and precipitation was obtained from the DWD/BfG-HYRAS v2.0 precipitation data set (Rauthe et al., 2013; Brienen et al., 2016). Hourly values for air vapor pressure deficit (VPD, haPa) were calculated after Goff and Gratch (1946) from recorded air temperature, humidity and air pressure using Watchdog 2700ET weather stations (Spectrum Technologies,Inc., USA) placed in a clearing close to the site at 2 m height. Volumetric soil water content (VSWC, %) was measured at 20 cm and 40 cm depth and at five points at each of the four sites, except in Bad Belzig, where it was measured at 20 cm and 60 cm depth due to the sandy soil. VSWC was measured using Teros 10 soil water content sensors (Meter group GmbH, Munich, Germany), connected to open-source data acquisition systems (“Loguino”, https://github.com/ArminNiessner/Loguino, last access: 15 December 2023). The five hourly time series at each site and soil depth were cleaned for outliers and obvious errors, and then averaged for each soil depth in order to receive the site’s average VSWC. Further analysis was carried out with data from 20 cm soil depth only, as the dynamic and values were very similar for the two soil depths (**Figure 2**). Historical data on the Soil Moisture Index (SMI), Soil Drought Intensity (SDI) and Magnitude (SDM) was extracted for each site from the German drought monitor (Zink et al., 2016) in order to rank the year of observation (2022) in the course of the last 70 years. The dataset enables drought estimates at a 4 x 4 km² resolution across Germany and gives estimates for the upper soil layer (25 cm) and total soil bulk.

### 2.3 Dendrometer measurements

At each location, ten apparently vital and dominant individuals of the same age and upper canopy level were selected for monitoring diurnal stem radial changes and sap flow (predominant or dominant, social class 1, Kraft, 1884). Two cores per tree of all trees were extracted before growth started in 2022, using a 5 mm increment borer (Haglöf, Sweden). Cores were used to determine tree age and mean annual growth over the previous ten years (2012–2021). Tree heights were determined using the “TruPulse Laser 200” (Laser Technology, Inc., USA). All selected individuals were equipped with spring-loaded linear displacement potentiometers (MMR 10 11 R5 K, MEGATRON Elektronik AG und Co., Munich, Germany) with a resolution of<10 µm to record stem radial changes. The potentiometers were mounted in the center of stainless steel frames and then attached to the trunk at a height of 1.3 m with two screws anchored at least 5 cm deep in the xylem and at least 6 cm from the measurement point. They were placed with their tips on the innermost bark after partially removing the outer parts without damaging living bark and cambium, and covered and shielded. Each dendrometer was connected to a “Loguino” attached to each tree and recorded every 10 minutes. Stem radial change (SRC) is the reversible and irreversible change in tree radius over time. Although tree-ring widths are not 1:1 comparable with SRC, as the latter includes additional bark tissue, we used the mean tree-ring width of the 10 previous years to put the total SRC of 2022 into context. The measured changes in radial stem dimension allowed for the calculation of the tree’s stem water deficit (TWD) following the method by Zweifel et al. (2016). The TWD is calculated as the difference between the highest previous stem radius and the current stem radius, given that the current stem radius is smaller than previously, otherwise, TWD = 0. Since we were interested in the soil moisture, below which the TWD can no longer be removed and persists over a longer period of time, we focused on the daily minimum TWD. This means that the daily TWD is only greater than 0, as soon as the TWD, which builds up during the day, cannot be removed overnight.

Unfortunately, we could only analyze four trees from HE and only two out of ten from ME as a result of a major dendrometer malfunction (9/10 for BB and RO). In total, 24 dendrometer sequences were available over all plots with recordings every 10 minutes.

### 2.4 Sap flow measurements

We used pairs of self-built sap flow sensors based on the method described by Granier (Granier, 1985, 1987). The sensors were installed at 1.3 m height on the north side of each trunk. The sensors consist of a pair of copper-constantan thermocouples placed inside 2 cm long needles in the xylem with about 10 cm vertical distance. The upper sensor is constantly heated with 120 mA and the temperature difference is recorded every 10 minutes with a “Loguino”, together with the dendrometer. Higher sap flow means faster heat dissipation and thus lower temperature differences. For each sensor, we calculated sap flow rates after Granier (Granier, 1985, 1987), taking the maximum temperature difference of each day and a linear interpolation in between as the corresponding reference value with zero sap flow. The obtained volume flux density of sap flow (in g cm^−2^ s^−1^) was further extrapolated for the whole tree, using the R package developed by Berdanier et al. (2016) which accounts for the tree’s sapwood area. Total sapwood area was obtained from visual inspection of tree cores from each tree.

### 2.5 Data analysis

Statistical analysis and data visualization were done using Python, version 3.10.13 (Van Rossum and Drake, 2009), with its packages NumPy v. 1.22.3 (Harris et al., 2020), pandas v. 1.4.2 (McKinney, 2010), SciPy v. 1.10.1 (Virtanen et al., 2020) and Matplotlib v. 3.7.1 (Hunter, 2007). We collected data from March until the end of October 2022. For our analysis, however, we were mainly interested in changes in sap flow and the persistence of TWD in relation to soil water content during the main growing season of 2022. All further analyses were carried out for the period from April 15 (DOY 106) to August 31 (DOY 244), as all trees had already started their radial growth from mid-April and as the sap flow measurements in HE had to be stopped at the end of August due to energy problems. Absolute values of sap flow rate and TWD can vary considerably as a result of varying tree dimensions and physiological parameters, even within species (Čermák et al., 1995; Brinkmann et al., 2016). Thus, we normalized the data by dividing the sap flow and TWD data of each tree by the average of the highest 2.5% maximum values of the respective tree in order to minimize the effect of single extreme values (analog to Brinkmann et al., 2016). The resulting values range from 0 to ∼1 and are the relative sap flow and relative TWD, respectively, and from here on will only be termed sap flow and TWD.

In order to compensate for differences in tree dimensions, we also calculated the basal area increment (BAI) and the relative BAI (rBAI), by calculating the areal increment in stem basal area according to the measured total annual SRC of 2022 and the proportion of this area in relation to the total stem basal area, respectively. Our first objective was to identify the onset of TWD during decreasing soil moisture content, i.e. identify the threshold in VSWC at which a TWD cannot be removed and persists, thereby inhibiting radial growth. However, VSWC is strongly dependent on soil conditions and therefore VSWC has been normalized in the form of relative extractable water (REW). REW represents the ratio between available soilwater and maximum extractable water (Granier, 1987) and is expressed in Equation 1 as:


(1) $$REW=\frac{VSWC-VSWC_{min}}{VSWC_{max}-VSWC_{min}}$$

where VSWC is the volumetric soil water content and assuming that ${\text{VSWC}}_{max}$ equals VSWC at field capacity and ${\text{VSWC}}_{min}$ equals the permanent wilting point. We therefore used the entire data set from March to October to calculate the REW. We plotted the daily minimum TWD of each tree against the respective site’s daily maximum REW at 20 cm depth and fitted the following exponential equation:


(2) $$TWD=a\;*\;e^{\left(b*REW\right)}$$

**Figure 3:**
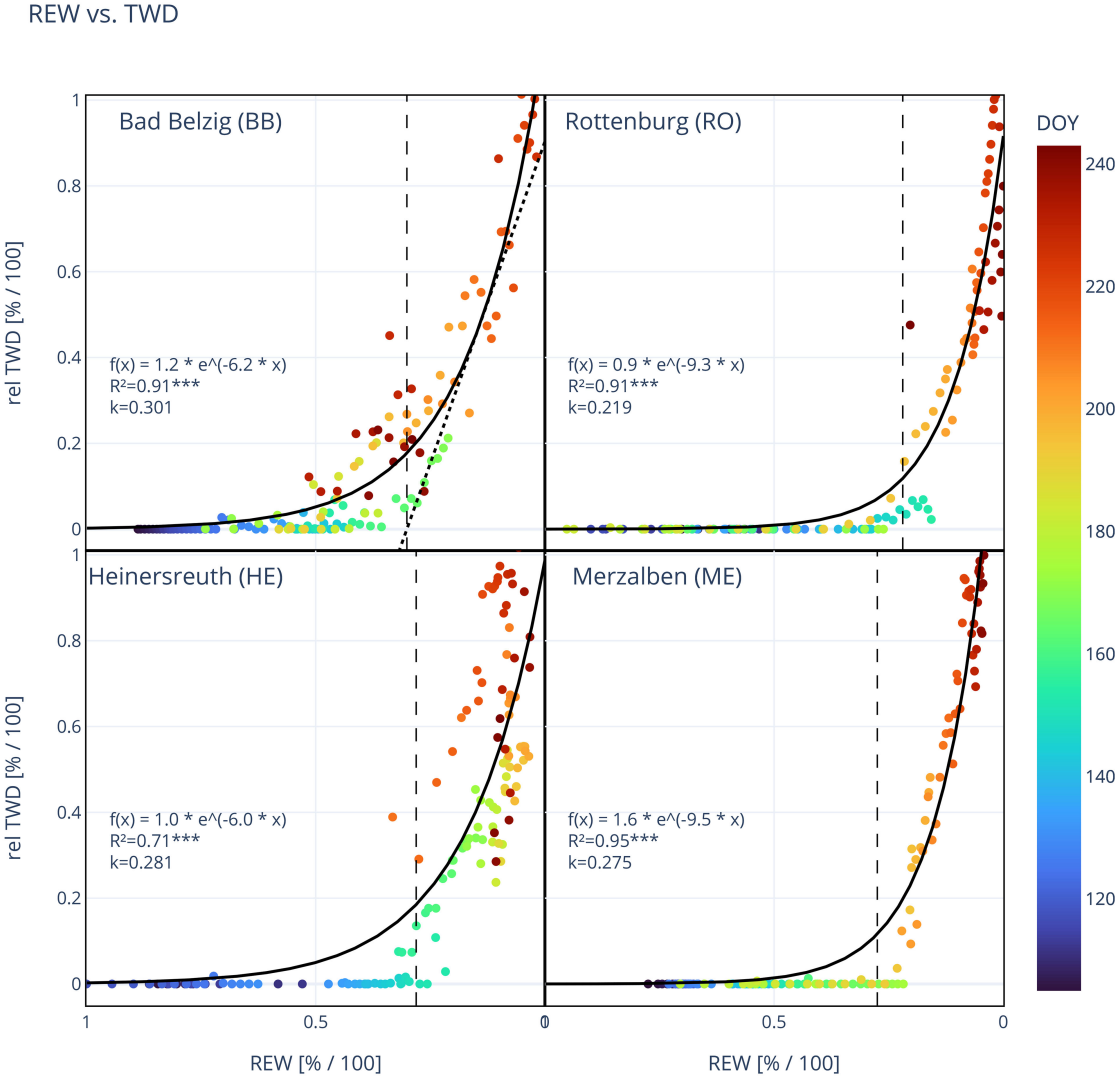
Site mean relative tree water deficit (rel TWD) in relation to relative extractable water (REW) at each site. Each dot represents a day and its color indicates the day of year (DOY 106–244). Solid line shows the exponential regression and vertical dashed line marks the threshold in REW (k), where a TWD is predominant. k is the intersection with the x-axis of the tangent to the exponential regression with a fixed slope, as exemplified by the dotted line in the upper left panel for BB (see also Materials and Methods). Function of exponential regression [f(x)], coefficient of determination (R², ***: p*<*0.001) and the value for k are shown in each panel. Number of trees observed: 9 (BB), 9 (RO), 4 (HE), 2 (ME).

Here, TWD is the relative daily minimum TWD, REW is the relative extractable water, and a and b are coefficients fitted for each tree individually and for the averaged time series of TWD for each site. We then calculated a tangent with a fixed slope using the “gradient”-function of the NumPy package. The intersection with the x-axis was defined as the threshold (k) at which TWD onsets. Analog to Brinkmann et al. (2016), we tested different fixed slopes (-2, -3, -4, -5) and decided to take -3 as it resulted in values for k that are close or identical to the value where one would intuitively expect it to be when looking at the plotted data (see also **Figure 3**, upper left panel). Although this is relatively subjective, it is still a reproducible method for determining the threshold value. Analog to this, we also calculated k for VSWC instead of REW, presented in **Supplementary Table S5**.

To analyze the changes in daily sap flow of trees to drying soils, we plotted the calculated daily sum of sap flow of each tree against the respective site’s REW at 20 cm depth. Dry air, i.e. a high VPD is a main driving force for sap flow (O’Brien et al., 2004) and under wet conditions (low VPD), sap flow is substantially reduced. We focused on the effect of soil drying on sap flow, therefore, we excluded days where maximum VPD did not exceed 1 kPa from the analysis. A linear regression was fitted to the data for REW in the range between 0 to 0.5. The slope m represents the percentage decrease in daily sap flow per percentage decrease in REW. Additionally, we used this linear regression to calculate relative sap flow at soil threshold k. Relative sap flow at maximum TWD was calculated taking the average relative sap flow for days with a relative TWD*>*0.9. Similarly, relative sap flow during maximum drought was calculated by taking the average relative sap flow for days where REW was lower than the 10th percentile, i.e. for the 10% lowest REW values. We performed a Tukey’s HSD test to check if the means of all measured and calculated tree parameters are statistically different between sites (p*<*0.05) using the “stats.tukey_hsd”-function of the SciPy package. A principal components analysis (PCA) was performed to identify the inter-relationship between the different tree variables and also between the calculated soil threshold k and the slope of the linear regression between REW and daily sap flow together with selected site variables. All variables were first standardized, setting the mean to zero and standard deviation to one in order to bring all variables within the same range.

## 3 Results

### 3.1 Meteorological conditions and tree responses

The year 2022 was one of the driest years within the last 70 years in terms of precipitation and soil moisture at all four sites (**Figure 1**; **Table 2**). Especially at BB, only 65% of the average long-term annual rainfall was recorded and the most intense soil drought since 1951. The year 2018 was generally the driest at all sites, except in ME, where 2011 was even drier. Exceptionally low rainfall was recorded between May and August in HE (38% of the long term rainfall during these months) and during July and Agust in ME (8.7%, **Figure 1**).

**Table 2:** The drought of 2022 compared to the long-term average annual rainfall (1986–2015), the soil drought intensity (SDI) and magnitude (SDM) recorded by the German drought monitor (1951–2022, https://www.ufz.de/droughtmonitor, last access: 15 December 2023, Zink et al., 2016).

	% of rain	SDI	SDM
BB	65	1.	2. (2018)
RO	84	2. (2018)	5. (2018)
HE	86	3. (2018)	8. (2018)
ME	75	4. (2011)	10. (2011)

The number in brackets indicates the year that ranked first, i.e. being the driest year recorded.

At site BB, especially from March to July rainfall was exceptionally sparse, leading to a decrease in VSWC in the course of the year (**Figure 2**). Growing season, i.e. radial growth, started between April 15–20. The first substantial TWD in most trees developed starting from June 2 and lasted until June 20, also leading to a noticeable stagnation in stem radial change (SRC) and a reduction in sap flow. A rainfall sum of 18 mm between June 19 and 20 recharged VSWC from 9.8% to 18.2% (from 21.1% to 74.1% in REW, June 21), allowing for a recovery of stem water reserves (TWD = 0) and leading to an almost instant onset of stem radial growth. Around July 10, again a substantial TWD developed in most of the trees while VSWC kept decreasing. Again, sap flow was strongly reduced and stem radius was actually decreasing over a period of over a month until August 17, where 22 mm of rain (August 13–18) recharged VSWC from 6.7% to 14.6% (from 1.8% to 52.2% in REW). TWD was removed, sap flow reached rates as before the drought period and stem radial growth recovered for a brief period of time until August 30. The following rainfall of less than 10 mm per day had almost no effect on VSWC but led to a partial removal of TWD in some trees, but no substantial radial growth was recorded onwards. Therefore, the average stem radial growth over the growing season was only 55% (1.39 mm) of the mean annual growth over the previous ten years (**Table 3**). Note that two trees (BB03 and BB06) were less affected, removing TWD much faster and showing higher growth rates, while at one tree (BB01) TWD remained on a high level until the end of observation.

**Table 3:** Average observed diameter at breast height (DBH), radial increment in 2022 estimated from dendrometer recordings (incr. 2022), mean tree ring width of the ten years before 2022 (TR 2012–2021), relative basal area increment (rBAI), mean and maximum tree water deficit (mean TWD and max. TWD), mean relative sapwood area (mean swa) and sap flow at the four sites.

	DBH [cm]	incr. 2022 [mm]	TR 2012- 2021 [mm]	rBAI [%]	mean TWD [µm]	max. TWD [µm]	mean swa [%]	mean sap flow [Kg day^−1^]	max. sap flow [Kg day^−1^]
BB	56.9 _10_ *^a^*	1.39 _9_ *^a^*	2.51 _9_ *^a^*	0.96 _9_ *^a^*	53 _9_ *^a^*	242 _9_ *^a^*	28.3 _10_ *^a^*	24.7 _10_ *^ab^*	55.5 _10_ *^ab^*
±	13.0	0.84	0.99	0.50	34.9	88.4	7.2	15.2	30.5
RO	38.9 _9_ *^b^*	2.87 _9_ *^b^*	3.78 _9_ *^b^*	2.92 _9_ *^b^*	105 _9_ *^b^*	537 _9_ *^b^*	47.3 _10_ *^b^*	40.8 _10_ *^b^*	91.5 _10_ *^b^*
±	4.9	0.45	0.67	0.58	39.6	159.6	4.9	14.9	27.6
HE	45.1 _10_ *^b^*	0.83 _4_ *^a^*	0.70 _4_ *^c^*	0.77 _4_ *^a^*	57 _4_ *^ab^*	205 _4_ *^a^*	25.7 _10_ *^a^*	17.9 _10_ *^a^*	39.7 _10_ *^a^*
±	7.3	0.50	0.27	0.38	34.3	72.7	7.9	20.2	35.0
ME	44.7 _10_ *^b^*	3.14 _2_ *^b^*	3.31 _2_ *^ab^*	2.83 _2_ *^b^*	54 _2_ *^ab^*	274 _2_ *^a^*	41.1 _10_ *^b^*	15.8 _10_ *^a^*	39.2 _10_ *^a^*
±	5.5	0.38	0.57	0.07	5.6	3.9	5.6	7.5	21.5

Superscript lowercase letters indicate groups that statistically differ from each other (p<0.05) and subscript numbers indicate the number of trees observed. The respective standard deviation is given beneath (± SD).

At RO, trees started to grow between April 12–15 (**Figure 2**). A first short drought started on May 25, where most trees developed a TWD and stagnated in radial growth. Sap flow was also noticeably reduced. Strong rainfall on June 4 and 5 (33 mm) recovered VSWC from 8.6% to 17.1% (form 15.7% to 72.5% in REW, June 6) and consequently, also reduced TWD and increased sap flow and radial growth. While VSWC started continuously dropping from July 1, on July 10 a point was reached where all trees started to develop a strong TWD and sap flow started to decrease. The following rainfall events during July and August were not enough to recover VSWC and fully recharge stem water reserves. TWD was reduced and sap flow increased again during the end of August and through September, but actual growth was terminated around July 10. The average increment of 2022 correspondents to 76% of the average increment over the previous ten years (**Table 3**).

At site HE, trees appear to have started growing in the first half of April, but substantially accelerated in growth during the first week of May (**Figure 2**). Over the course of the year, VSWC continued to decrease and a TWD started to develop around June 1. This led to an instant stop in radial growth and sap flow continuously decreased. Only tree HE01 continued to grow until the second half of July when a TWD developed and growth started to stagnate. Exceptionally heavy rainfall during September allowed for a partial removal of TWD and an expansion in radius, but then stayed at this level with no further growth. The average total annual increment at HE was only 0.83 mm or 0.59 mm, if HE01 is excluded. Tree ring widths over the previous ten years are generally small, so it corresponds to 19% more growth or 16% less growth, respectively (**Table 3**). Accounting for the bark tissue included in the increment of 2022, it would be less.

At ME, the onset of radial growth was between April 12–15 and continued in a linear manner until an intense TWD developed which ceased growth and reduced sap flow (**Figure 2**). VSWC continuously decreased in the course of the year, only shortly interrupted by 40 mm of rain between June 23–26, until a rainy period in September recharged VSWC and allowed for a removal of TWD around September 9. This led to a radial stem recovery but no further growth. Mean total annual increment was 3.14 mm which is only slightly less (95%) than the average annual increment over the previous ten years (3.31 mm) of the two analyzed trees.

### 3.2 Effect of drying soils on tree water deficit and sap flow

A decrease in soil moisture, expressed as the relative extractable water (REW), below a certain value induced an exponential increase in TWD, indicating a threshold in REW (and also VSWC) where TWD persits. TWD in relation to REW is represented by an exponential regression for all individual trees (not shown) and site mean TWD (**Figure 3**). Coefficients of determination for individual trees (BB: 0.82 ± 0.10, RO: 0.90 ± 0.04, HE: 0.58 ± 0.20, ME: 0.95 ± 0.01, **Supplementary Tables S1–S4**) and for site means (BB: 0.91, RO: 0.91, HE: 0.71, ME: 0.95) were very high and statistically significant (p*<*0.001). The distribution of values and the shape of the exponential regressions were highly similar within and also between sites, however, slightly shifted along the x-axis. This resulted in k-values that are within a small range at one site but at different levels at different sites. Especially at RO, k was statistically lower compared to BB and HE (p*<*0.05) and also ME (p=0.128, **Table 4**). Coloring the data points by their day of year revealed that dry days with low REW occurred mainly during the second half of the vegetation period (orange and red dots) while during the first period TWD stayed mainly close to zero (dark and bright blue). This also illustrates that on some days early that year a TWD was avoided even below our estimated k, but later that year a REW above k even led to a TWD, suggesting a slight shift of k during the year, i.e. drought conditions that induce a TWD were already reached at higher values for REW. Higher values of maximum daily VPD are more frequent during the second half of the year (**Figure 2**) and are therefore also associated with a higher TWD, but showed no clear relationship (**Supplementary Figure S2**).

**Table 4:** Average threshold in REW where TWD persists (k), coefficient of determination (R^2^) for exponential regression between TWD and REW, slope of linear regression between sap flow and REW (m), for REW*<*0.5, and respective coefficient of determination (R^2^), mean relative sap flow at threshold k, mean relative sap flow at the 10% lowest REW, i.e. at maximum drought and mean relative sap flow at a relative TWD*>*0.9, i.e. maximum TWD.

	k [%]	R^2^ REW/ TWD	m [%]	R^2^ REW/ sap flow	sap flow at k [%]	sap flow at max drought [%]	sap flow at max TWD [%]
BB	29.6 _9_ *^a^*	0.82 _9_	1.3 _10_ *^ab^*	0.66 _10_	43.5 _9_ *^a^*	17.1 _10_ *^a^*	16.2 _9_ *^a^*
±	3.6	0.10	0.4	0.19	10.4	12.9	13.0
RO	21.7 _9_ *^b^*	0.90 _9_	1.1 _10_ *^b^*	0.62 _10_	43.9 _9_ *^a^*	18.1 _10_ *^a^*	13.5 _9_ *^a^*
±	1.6	0.04	0.2	0.12	8.6	6.2	4.4
HE	27.3 _4_ *^a^*	0.58 _4_	1.6 _9_ *^ac^*	0.69 _9_	48.9 _4_ *^a^*	16.5 _9_ *^a^*	15.9 _4_ *^a^*
±	5.0	0.20	0.3	0.11	2.5	10.6	6.5
ME	27.5 _2_ *^ab^*	0.95 _2_	2.0 _10_^c^	0.82 _10_	53.5 _2_ *^a^*	8.6 _10_ *^a^*	3.6 _2_ *^a^*
±	1.3	0.01	0.5	0.17	4.5	7.1	0.1

Superscript lowercase letters indicate groups that statistically differ from each other (p<0.05) and subscript numbers indicate the number of trees observed. The respective standard deviation is given beneath (± SD).

**Figure 4:**
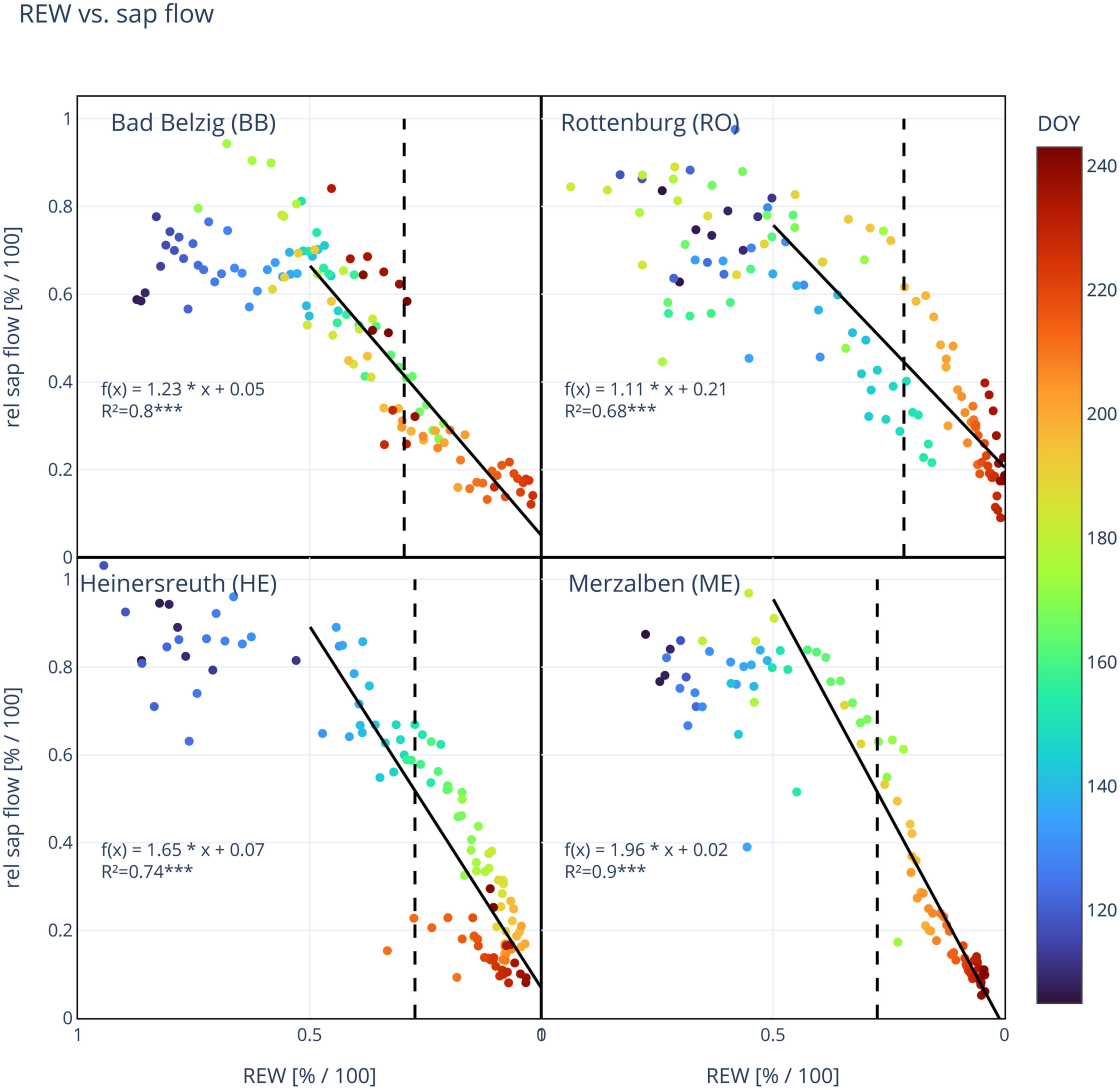
Site mean relative sap flow in relation to relative extractable water (REW). Each dot represents a day and its color indicates the day of year (DOY 106–244). Solid line shows the linear regression for REW*<*0.5 and vertical dashed line marks the threshold in REW (k), where a TWD is predominant. Function of linear regression [f(x)] and coefficient of determination (R², ***: p*<*0.001) are shown in each panel. Number of trees observed: 10 (BB), 10 (RO), 10 (HE), 10 (ME).

Relative sap flow per day started to decrease with drying soils between 50 and 40% REW and can be described with a linear regression (**Figure 4**). Coefficients of determination were relatively high for each individual tree (BB: 0.66 ± 0.19, RO: 0.62 ± 0.12, HE: 0.69 ± 0.11, ME: 0.82 ± 0.17, **Supplementary Tables S1–S4**) and for site mean sap flow (BB: 0.80, RO: 0.68, HE: 0.74, ME: 0.90), all highly significant (p*<*0.001). We observed statistically significant differences between the decrease in sap flow and decrease in REW (slope m of linear regression), but not between the relative sap flow at threshold k and at lowest occurred REW for all trees at all sites (**Table 4**). The daily maximum VPD showed little effect on daily sap flow (**Supplementary Figure S3**). For REW*<*0.5, relative sap flow decreased by 1.1 ± 0.2% in RO to 2.0 ± 0.5% in ME per 1% decrease in REW, suggesting a stronger reduction in sap flow to soil drying at more oceanic/humid sites.

### 3.3 Inter-relationship of tree and site variables

A PCA on a selection of eight tree variables (**Table 5**) shows that maximum TWD, relative basal area increment (rBAI), relative sapwood area (swa) and mean tree ring width from 2012 to 2021 (TR 2012–2021) are positively related with each other and mainly loaded on axis 1, which explains about 52% of the variance. The threshold k is also mainly loaded on axis 1 but negatively related to the before mentioned tree variables. Axis 2 explains about 19% of the variance and mainly represents DBH and mean sap flow, being positively correlated. The slope of the linear regression between daily sap flow and REW (m) is mainly loaded on axis 3 (12% explained variance) and showing a slight positive relation to DBH, k and TR 2012–2021.

**Table 5:** Principal components analysis (PCA) of eight selected tree variables.

Variables	Axis 1	Axis 2	Axis 3
	(0.525)	(0.189)	(0.125)
DBH	-0.177	**0.709**	0.258
max. TWD	**0.403**	0.083	0.010
k	**-0.387**	0.140	0.211
m	-0.123	-0.269	**0.880**
mean sap flow	0.348	**0.460**	-0.002
rBAI	**0.438**	-0.229	0.128
swa	**0.421**	-0.173	0.177
TR 2012–2021	**0.390**	0.323	0.259

Given are the loadings of each variable along the three most important axes. The eigenvalues are indicated in brackets and quantify the amount of variance captured. Numbers in bold indicate a close relationship of that variable to the respective axis. Variables that show high loadings along the same axis and the same direction are positively correlated with each other and negatively if in opposite direction.

**Table 6** shows the PCA for the soil threshold k and the slope of the regression between REW and mean daily sapflow m together with five selected site variables. Axis 1 explains 55% of the variance and encompasses in one direction mainly m, long-term annual rainfall and depth of soil where 90% of fine-roots where found, while the percentage of fine-roots encountered within the upper 25 cm is loaded in the opposite direction. The soil threshold k is mainly loaded on Axis 2 (25% of variance) together with the site basal area and opposite to the site long-term rainfall. The number of stems per hectare is mainly represented by Axis 3 (13% of variance) and negatively related to k and m.

**Table 6:** Principal components analysis (PCA) of k, m and five selected site variables.

Variables	Axis 1	Axis 2	Axis 3
	(0.554)	(0.251)	(0.131)
k	0.076	**0.651**	-0.372
m	**0.402**	0.053	**-0.457**
lt. rainfall	**0.415**	**-0.412**	-0.015
BA	0.385	**0.447**	-0.027
stems per ha	0.210	0.348	**0.799**
FR<25 cm	**-0.502**	0.024	-0.111
FR depth	**0.464**	-0.286	0.012

Given are the loadings of each variable along the three most important axes. The eigenvalues are indicated in brackets and quantify the amount of variance captured. Numbers in bold indicate a close relationship of that variable to the respective axis. Variables that show high loadings along the same axis and the same direction are positively correlated with each other and negatively if in opposite direction.

## 4 Discussion

### 4.1 Response of TWD to soil drying

The relative daily minimum tree water deficit (TWD) of Douglas-fir shows a strong non-linear response to soil drying (**Figure 3**). Interestingly, this non-linear response was within a small range of relative soil water availability (REW) for all trees at each site, indicating a site-specific threshold (k) in REW. Below this threshold, the daily occurring TWD could not be removed overnight anymore, but rather accumulated over time. Also, Brinkmann et al. (2016) reported a threshold for four temperate tree species (*Fagus sylvatica*, *Picea abies*, *Acer pseudoplatanus* L. and *Fraxinus excelsior* L.) at Lägeren, Switzerland. Surprisingly, this threshold was not different between species. In contrast to Brinkmann et al. (2016), we used the daily minimum relative TWD instead of the midday maximum TWD, as we wanted to study when the daily occurring TWD due to transpiration and following discharge of stem water reserves, cannot be recharged overnight as a consequence of declining soil moisture. We also took REW instead of VSWC in order to compare the different sites with differing soil conditions. Especially at HE, the absolute VSWC level was almost twice as high compared to the other sites and also reversed in its moisture gradient (see **Figure 2**). The average soil threshold k was lowest in RO (21.7%) and significantly higher at the three other sites (27.3%–29.6%, **Table 4**). The results of a PCA between the different tree variables suggest a negative relationship between k and maximum TWD, relative basal area increment, as well as relative sapwood area (**Table 5**). The sapwood cross-sectional area scales with the relative amount of stored water used for daily transpiration (Goldstein et al., 1998; Phillips et al., 2003; Meinzer et al., 2004). However, reversible stem radius changes are largely attributable to the inner bark (Zweifel et al., 2001), but both the sapwood depth and inner bark thickness are correlated with the leaf area (Gartner, 2002). Therefore, a larger relative sapwood area due to a small DBH and a deep sapwood corresponds to a larger inner bark thickness, allowing for larger reversible stem radius changes and thereby a larger mean and maximum TWD, as observed at RO. According to different studies on conifer species (e.g. Sellin, 1996 and Galván et al., 2012), radial growth and BAI scales with sapwood area, which our results also suggest. A large growth rate and sapwood area like the trees in RO, could also be linked to a better drought resistance, represented as a low soil threshold k. For example, either by ensuring that the water potential in the inner bark and cambium reaches a correspondingly low level, which allows sufficient hydration of the cambium even with low REW and thus also enables radial growth. Or by a higher radial growth also being accompanied by a stronger root growth and thus additional water sources can be easier accessed.

Site characteristics also might influence the threshold k, as trees respond and adapt to their growing conditions. A high competition between trees, represented by a high number of stems and basal area (BA), appears to be positively related to k. However, one would expect that a high competition leads to a more rapid depletion of soil water reserves rather than affecting the threshold k (Belmonte et al., 2022). In contrast, the long-term annual precipitation is negatively related to k, meaning that at drier sites, the trees tend to experience drought already at higher levels of soil water potential, represented as relative extractable water (REW) available. A deeper rooting depth also appears to be weakly related to the threshold k. This would be reasonable, as deeper rooting also provides a larger water reservoir accessible. While the upper soil layer is already very dry, a persistent TWD can still be prevented by shifting water uptake to deeper soil layers, as e.g. reported by Brinkmann et al. (2019) for different temperate tree species. However, the volumetric soil water content from 20 and 40 (60) cm depth tends to decrease with increasing soil depth, with the exception of HE.

Even if the response of the individual trees within a site was very homogeneous, there were a few trees that deviated from this. In BB, two individuals, especially BB03, showed significantly more growth, partly due to a faster reduction of TWD and a slightly lower threshold k (**Figure 2**; **Supplementary Table S1**). Likewise in HE, one tree (HE01) grew significantly more than the other three studied trees and had the lowest value for k. It could be that these trees have a larger water reservoir available to them due to topographical or geological features, such as local depressions and faults which trap water, but this is pure speculation without more detailed investigations of the underground conditions.

Using REW at 40 cm (60 cm at BB) soil depth resulted in almost the same values for k, except at HE, where VSWC at 40 cm soil depth was generally higher than at 20 cm. Here, the calculated average threshold for a TWD was 34.3% at 40 cm soil depth, compared to 27.3% at 20 cm. Sites HE and RO are characterized by a clay layer starting at around 50 cm soil depth. While the terrain at RO is inclined and therefore well drained, HE is rather flat and watterlogging may appear, which was observed in December 2023. According to Kutschera and Lichtenegger (2002), the depth of growth of the roots is greatly reduced on very wet, waterlogged soils and such sites are therefore not so suitable for Douglas-fir trees. Waterlogging induces damage to the fine root system of Douglas-fir (Lavender and Hermann, 2014) and reduces P-uptake by mycorrhiza (Gadgil, 1972). Rout and Sahoo (2015) found that under waterlogged and acidic soil conditions, iron may be taken up excessively leading to damages of vital cellular constituents in plants. This root damage reduces water uptake efficiency and may increase the threshold k.

According to a study from Warren et al. (2005), water extraction shifts to deeper layers during prolonged summer droughts. However, daily water uptake from the entire 2 m profile was strongly dependent on the water potential at 20 cm, suggesting that fine roots in the topsoil may play an important role in regulating water uptake through hydraulic effects on stomatal conductivity. A systematic error in VSWC’s measurements can be ruled out, as measurements were taken at five different locations with five separate data loggers at each site, and the measured values at all five locations deviated only slightly from each other (standard deviation of VSWC at 20 cm soil depth between positions per site; BB: 4.4%, RO: 3.0%, HE: 1.7%, ME: 3.0%). Defining a different gradient for the tangent (e.g. -2 or -4, instead of -3) in order to determine k does not significantly change the results, at least as long as the value is within a reasonable range. Less subjective would be a segmented or piecewise linear regression (Pilgrim, 2021), which we also tested for comparison at a later stage. We determined the intercept with the x-axis of the steep segment of the linear regression as the threshold in REW, which resulted in the following k values for the four sites (BB, RO, HE and ME): 31%, 19%, 33% and 24%. These values are very similar to the values determined using the tangent with the fixed slope, whereby in RO the threshold k is possibly further underestimated and in HE overestimated. For BB and ME, however, this method could be more accurate.

### 4.2 Responses of sap flow to soil drying

The relative daily sap flow of Douglas-fir shows a strong linear decrease in response to drying soils below 50% REW. Relative daily sap flow decreased between 1.1–2.0% per 1% decrease in REW, while being significantly lower at RO than at HE or ME, where m was largest. A stronger decrease in relative daily sap flow could mean that a high daily sap flow can be maintained until lower values of REW until stomata start to close due to soil drought conditions. At HE and ME, a notable decrease in daily sap flow starts around 40% REW, while at BB and RO, already at around 50% (**Figure 4**). However, this decrease in sap flow (m) appears to shift in RO during the year. During the first half of the year (DOY*<*180) sap flow starts to decrease at around 50%, leading to a lower decrease in sap flow per decrease in REW, while later on a notable decrease in sap flow starts at around 40% REW and being much steeper (m = 1.7, see **Supplementary Figure S3**).

The decrease in daily sap flow m is mainly represented by axis 3 in our PCA, which only explains 12.5% of the variance and is poorly linked to the other selected tree variables (**Table 5**). Site variables like long-term precipitation and rooting depth seem to be closer related to m (**Table 6**). Trees at more continental/drier sites might be adapted to longer dry periods and therefore start to conserve water already at higher levels of REW, while at more oceanic/humid sites, trees only rarely experience extended periods of drought. A deeper rooting and therefore probably a better access to water resources should allow for maintaining a high daily sap flow at REW levels below 50% and resulting in a steeper decrease in sap flow below this. The relative amount of fine roots encountered in the upper soil layer (soil depth<25 cm), however, was negatively related to m and might be a result of its inverse relation to rooting depth. While the relative root number generally decreased with increasing soil depth at BB, RO and HE, the distribution of fine roots was more evenly along the soil profile at ME, where rooting depth was also deepest (**Supplementary Figure S1**).

Other studies mainly compared the daily sap flow with the volumetric soil water content (VSWC) instead of the REW, so we also determined the linear decrease in sap flow due to soil drying expressed as VSWC (**Supplementary Figure S2**; **Supplementary Table S5**). At all four sites, relative daily sap flow decreased by an average of 5.66% (SD: ± 1.58%) per 1% decrease in VSWC, with no statistically significant difference between sites.

A comparison with other tree species and locations reveals a broad spectrum, with sap flow decreasing by 3.9–14.1% per percent decrease in soil moisture: Slow decreases were reported in Tyrol, Austria with 5.2% for *Pinus sylvestris*, 4.3% for *Picea abies* and 4.0% for *Larix decidua* (Leo et al., 2014). Hölscher et al. (2005) examined trees in Germany at two different locations and measured a reduction in sap flow of 3.9% for *Fagus sylvatica* on the first site, and on the second site the reduction was 9.3% for *Carpinus betulus* L., 10.9% for *Tilia cordata* Mill. and even 11.3% for *Acer pseudoplatanus*. A strong reduction was measured in the semiarid northwestern China with 14.1% for *Picea crassifolia* Kom (Chang et al., 2014).

This general linear decrease over a certain range of VSWC probably reflects a linear reduction in stomatal conductance due to closing stomata to conserve water and to avoid critically low water potentials that would put a tree at risk of hydraulic failure (Schulze and Hall, 1982). Only at very low REW (bottom right in each panel of **Figure 4**), the relative reduction in daily sap flow looks to be exponential, which might reflect a complete closure of stomata close to the permanent wilting point or even the occurrence of embolized vessels, sharply decreasing water conductance through the xylem.

Brinkmann et al. (2016) reported a decrease of 33% for *Picea abies*, however, for maximum midday sap flow, and a maximum reduction by 92% in response to decreasing soil moisture.

Trees at all four sites maintained on average almost half of their relative daily sap flow (45.4%, SD ±8.7%) at their individual threshold k where TWD could not be eliminated anymore. Even during maximum soil drought, and correspondingly maximum TWD, still on average 15.0% (SD ±9.9%) and 14.1% (SD ±9.1%), of relative daily sap flow was maintained, respectively (**Table 4**). This shows how water potentials in the canopy can reach much lower values than in the bark. Maintenance of leaves and reproduction in the canopy is clearly prioritized over secondary growth. This linear reduction of sap flow and the maintenance of sap flow at k and maximum drought seem to be species-specific, at least in the temperate zones of Central Europe, but also subject to a certain adaptation along precipitation gradients within a species, such as for Douglas-fir.

The mean and maximum absolute water consumption ranged from 15.8 to 40.8 and 39.2 to 91.5 kg day^−1^, respectively, and is similar to that reported by others for Douglas-fir (Fritschen et al., 1973; Granier, 1987) and other temperate tree species (Wullschleger et al., 1998; Hölscher et al., 2005). Except from ME, the mean and maximum daily water consumption was positively scaled with the relative sapwood area (swa, **Table 3**).

### 4.3 Soil drought causes early growth cessation

For most Douglas-fir trees in 2022, the growing season was effectively already over by mid-July (June in HE) as a consequence of a persistent TWD caused by soil drought. Based on our own experience and the limited literature we found about the end of the growing season for Douglas-fir in Central Europe (Miller et al., 2022), radial growth normally lasts until September or even October if temperatures and moisture availability are favorable. The “zero growth concept” posits that stem radial growth, governed by turgor and water potential conditions in the cambium (Lockhart, 1965; Steppe et al., 2006), becomes nearly impossible during prolonged TWD periods (spanning several days) with a bias of 1–5% (Zweifel et al., 2016). This cut in half of the growing season resulted in significantly less radial growth in BB and RO compared to the average growth of the previous ten years (**Table 3**). Growth was only slightly reduced in ME, while interestingly in HE, growth was around the previous average if accounting for bark tissue included in the total SRC of 2022. In addition, the mean value in HE is based on only 4 trees, one of which showed significantly more growth. Without tree HE01, the mean annual increment would be only 0.59 mm, i.e. approx. 84% of the mean annual ring width.

Particularly in RO, a TWD of 52–321 µm persisted at the end of observation (October 20) and through winter, for some trees even until the start of the growing season 2023, although VSWC (REW) recovered well above k in November and December (data not shown). This might reflect how newly built cells by the cambium around July 10, still turgescent at this time, dehydrated in the course of the following TWD, while cell walls matured and stiffened, which normally take several days (e.g. 7–17 days for Norway spruce, Anfodillo et al., 2012). This would result in an apparent TWD in the bark, although cells are turgescent, but due to cell maturation in a partly dehydrated state, the previous maximum radial extension cannot be reached by rehydration alone.

Plants are known for their phenotypic plasticity and adaptability to their environment (DeWitt and Scheiner, 2004) and also Douglas-fir can adapt to drought (Martinez-Meier et al., 2009). Therefore, we hypothesized that the trees at the driest site BB are the most tolerant to drought and at ME the most sensitive, based on the mean annual precipitation received at the site. However, in terms of the threshold k, our data show only a weak link to the long-term site precipitation (**Table 6**) and k does not differ significantly between the most continental/driest (BB) and the most oceanic/humid site (ME).

The reduction in sap flow due to increasing soil drought follows the gradient in humidity, being significantly stronger at the most oceanic/humid site ME. Here, the start of the decrease is shifted to about 40% REW and also heaviest reduced at maximum drought and maximum TWD. Granier (1987) even reports that transpiration of Douglas-fir at a site close to Nancy with oceanic character started to reduce sap flow at a REW of as low as 30%. As mentioned before, this could be interpreted as an adaptation to the low frequency of extended drought periods at sites with an oceanic character, allowing for a lower safety margin. At continental sites, on the contrary, droughts are usually more frequent and longer, making it more important to conserve water under drying soils. This would suggest a plastic adaptation of the trees to drier conditions, however, the gradient we studied across Germany is rather small and a study on a larger gradient across Europe would be more revealing. In addition, there may also be seasonal differences within a site, as could be observed in RO, where the decrease in relative sap flow later in the year was more similar to that at more oceanic sites.

Contrary to our expectations, the reduction in increment compared to the tree ring widths of the previous 10 years was strongest in BB with 55%, while in ME, the most oceanic site, the reduction in increment was only slight and in HE the increment in 2022 was even higher. However, as mentioned before, taking in mind that tree ring widths are not 1:1 comparable to dendrometer measurements, as the later also includes bark tissue, and that the mean increment for HE was skewed by one individual.

An important characteristic of Douglas-fir in connection with the expected climate changes is its greater tolerance to summer drought and faster recovery from drought years than other species in a variety of locations and under different climatic conditions (Vitali et al., 2017; Miller et al., 2023). However, the increasing dehydration of the soil and summer drought is also increasingly affecting Douglas-fir trees and may also make it more susceptible to existing and future pests. This could jeopardize the future viability of Douglas-fir as a replacement for Norway spruce. Our study so far is based on only one year, which was also exceptionally dry, but presumably representative for future years. The following years will show whether the trees are able to compensate for the drought stress in 2022, and remain vital, or whether they will even still struggle with legacy effects of the summer drought of 2018.

## Data availability statement

The datasets presented in this study can be found in online repositories. The names of the repository/repositories and accession number(s) can be found below: https://osf.io/df5va/.

## Author contributions

AN: Data curation, Formal analysis, Investigation, Methodology, Software, Visualization, Writing – original draft, Writing – review & editing. SE: Formal analysis, Investigation, Methodology, Visualization, Writing – original draft, Writing – review & editing. RZ: Conceptualization, Investigation, Methodology, Resources, Validation, Writing – review & editing. VH: Conceptualization, Investigation, Methodology, Validation, Writing – review & editing. DR: Investigation, Methodology, Writing – review & editing. AL: Conceptualization, Data curation, Funding acquisition, Investigation, Writing – review & editing. GS: Investigation, Methodology, Validation, Writing – review & editing. SH: Conceptualization, Funding acquisition, Project administration, Supervision, Writing – review & editing.
