# Supplementary material for: Soil drought sets site specific limits to stem radial growth and sap flow of Douglas-fir across Germany
Source: Front Plant Sci. 2024 Aug 6;15:1401833. doi: 10.3389/fpls.2024.1401833 (PMC11333354; doi:10.3389/fpls.2024.1401833)
Supplement: Supplementary file 1 [file DataSheet_1.pdf]

Supplementary Material

1 FIGURES

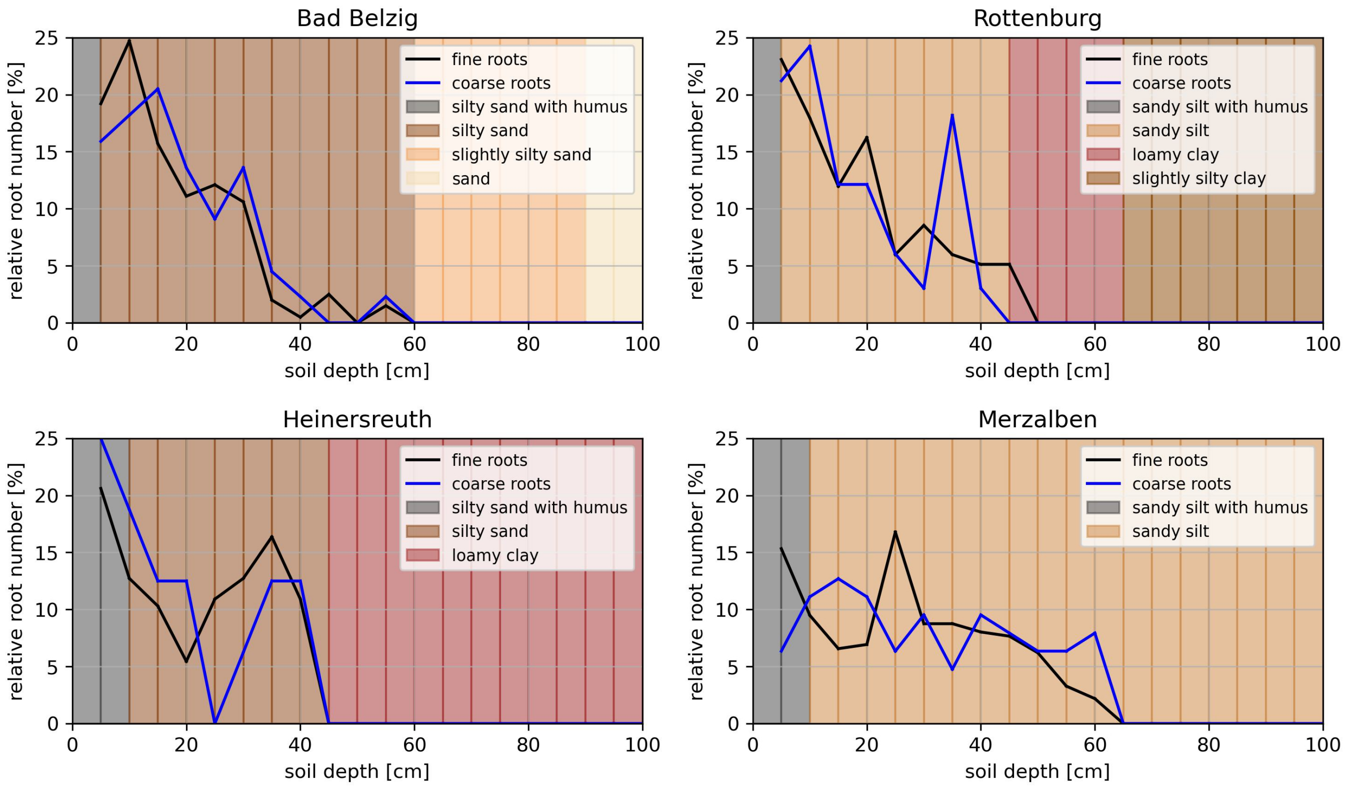

Figure S1. Soil texture and distribution of coarse (diameter>2mm) and fine (diameter<2mm) roots up to 100 cm soil depth.

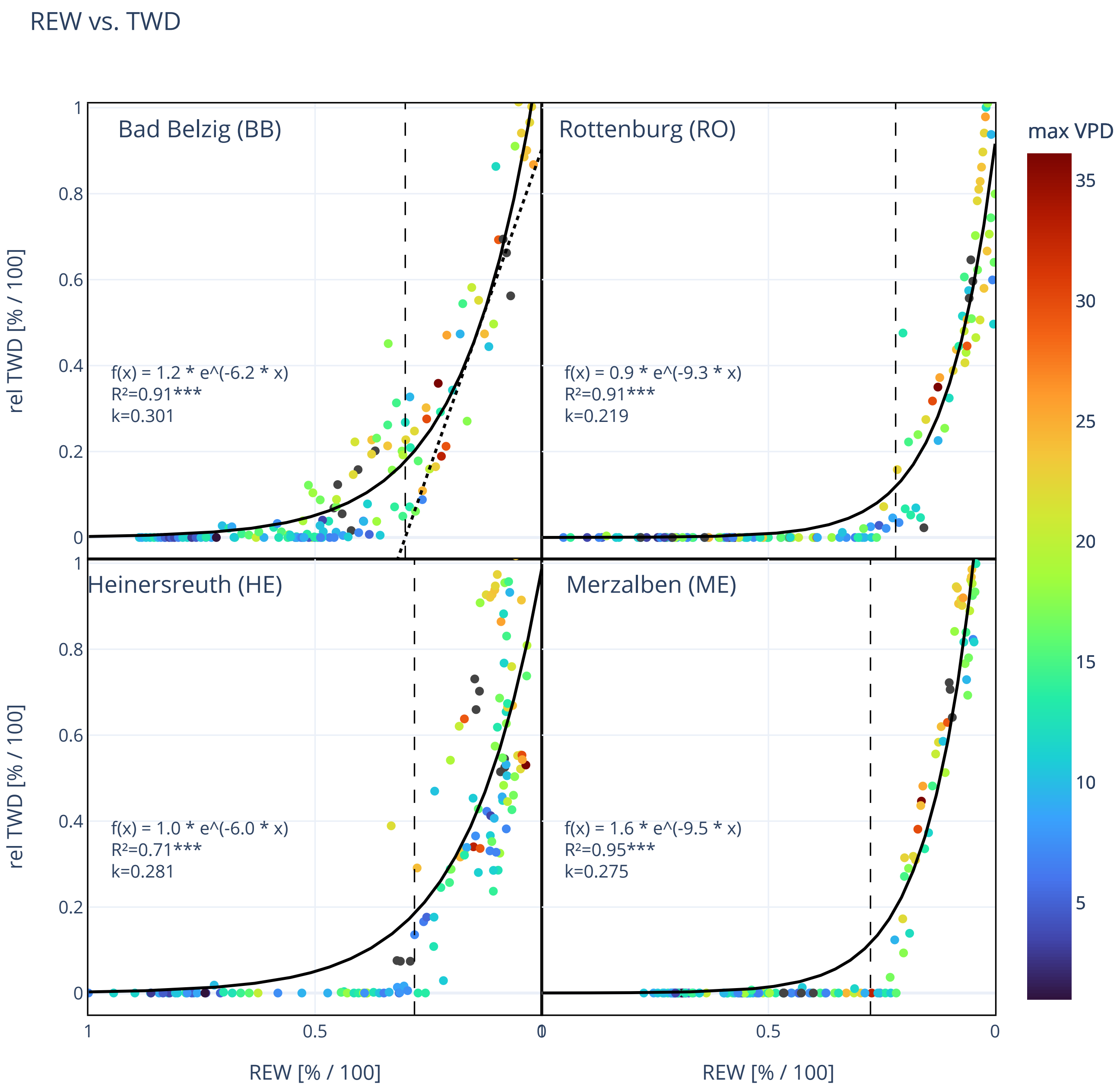

Figure S2. Site mean relative tree water deficit (rel TWD) in relation to relative extractable water (REW) at each site. Each dot represents a day and its color indicates the maximum air vapor pressure deficit (VPD) of that day. Solid line shows the exponential regression and vertical dashed line marks the threshold in REW (k), where a TWD is predominant. k is the intersection with the x-axis of the tangent to the exponential regression with a fixed slope, as exemplified by the dotted line in the upper left panel for BB (see also Material and Methods). Function of exponential regression (f(x)), coefficient of determination (R^2, ***: $p<0.001$) and the value for k are shown in each panel. Number of trees observed: 9 (BB), 9 (RO), 4 (HE), 2 (ME).

REW vs. sap flow

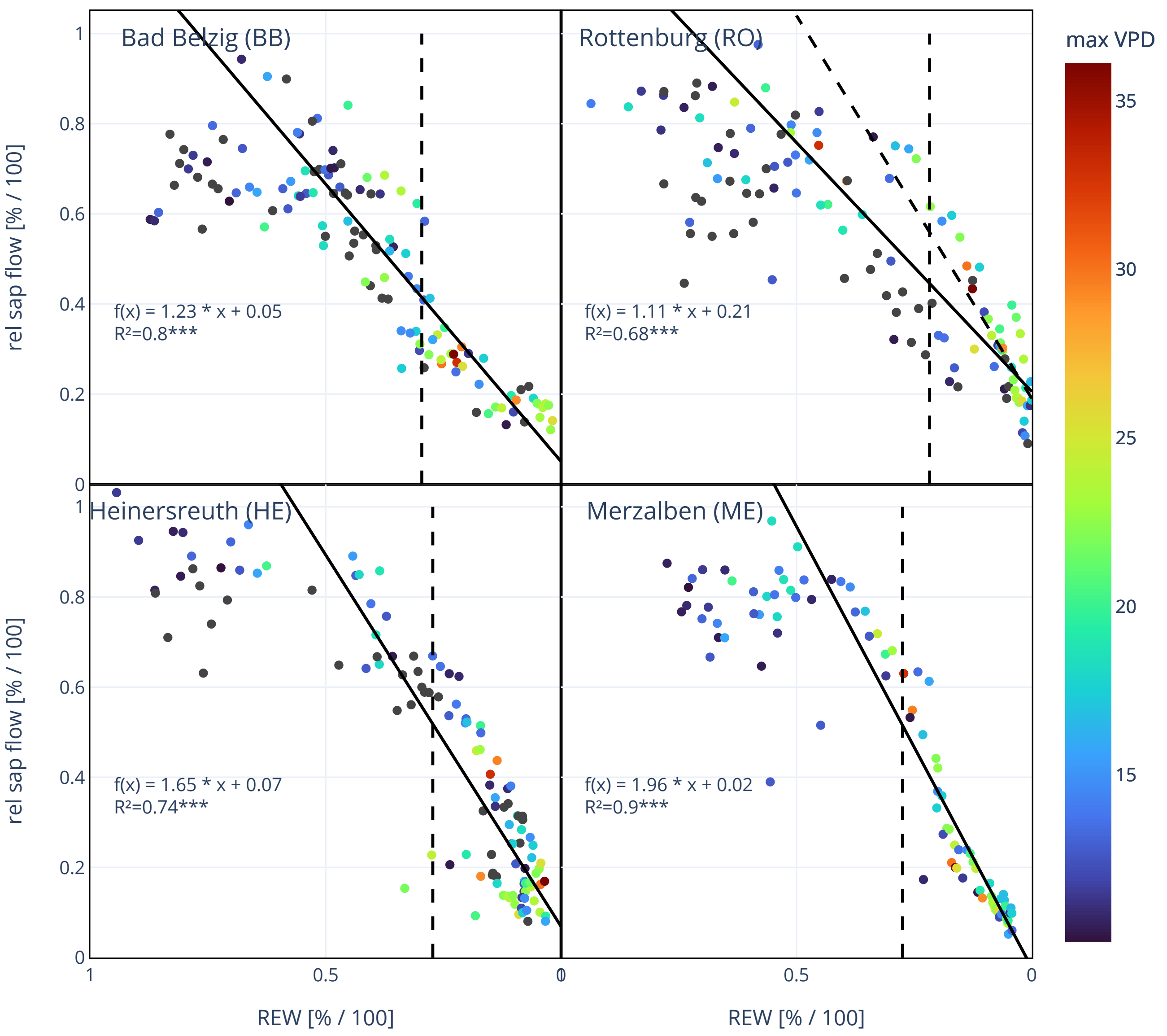

Figure S3. Site mean relative sap flow in relation to relative extractable water (REW). Each dot represents a day and its color indicates the maximum air vapor pressure deficit (VPD) of that day. Solid line shows the linear regression and vertical dashed line marks the threshold in REW (k), where a TWD is predominant. Function of linear regression (f(x)) and coefficient of determination (R^2, ***: $p<0.001$) are shown in each panel. For RO (top right panel), an alternative linear regression is shown as a dashed line for the period between 1 July and 31 August (f(x) = 1.7 * x + 0.19). Number of trees observed: 10 (BB), 10 (RO), 10 (HE), 10 (ME).

2 TABLES

Table S1. Tree characteristics at Bad Belzig (BB). Diameter at breast height (DBH), radial increment in 2022 estimated from dendrometer recordings (incr. 2022) and mean tree ring width of the ten years before 2022 (TR 2012-2021). For the period from April 15 to August 31, 2022: mean and maximum tree water deficit (mean TWD and max. TWD), threshold in soil water content where TWD persists (k) and coefficient of determination (R^2 [1]) for exponential between relative extractable water (REW) and TWD. Depth of the sap wood (sw depth) and relative sap wood area (swa). Mean sap flow (sf), slope of linear regression between REW and sap flow (m), its coefficient of determination (R^2 [2]), relative sap flow at threshold k (sf at k), sap flow at maximum drought (sf at max dr.) and sap flow at maximum TWD (sf at max TWD).

BB	tree height [m]	DBH [cm]	incr. 2022 [mm]	TR 2012–2021 [mm]	max. TWD [μm]	mean TWD [μm]	k [%]	R^2 [1]	sw depth [cm]	swa [%]	mean sf [Kg day^{-1}]	m [%]	R^2 [2]	sf at k [%]	sf at max dr. [%]	sf at max TWD [%]
01	33.8	53.5	1.59	2.41	402	141	34.6	0.63	4.30	29.6	21.4	1.5	0.82	48.9	6.3	4.3
02	34.0	47.8	0.81	2.15	194	29	29.5	0.93	4.60	34.8	19.5	1.6	0.79	43.2	14.0	13.6
03	33.0	68.8	3.41	3.28	227	33	22.9	0.84	6.55	34.5	57.2	1.4	0.76	49.0	20.8	19.9
04	28.5	42.7	1.15	2.44	217	42	29.1	0.91	3.20	27.7	5.9	0.8	0.55	28.9	17.3	19.7
05	34.2	52.9	0.50	0.97	146	39	29.4	0.73	3.65	25.7	12.1	1.5	0.65	59.7	26.2	22.1
06	32.0	43.9	1.60	1.23	217	48	29.7	0.75	2.0	17.4	18.5	0.7	0.22	53.0	48.5	45.2
07	33.8	58.4	0.99	2.65	142	28	25.6	0.89	5.65	34.9	37.2	1.9	0.88	42.1	8.6	5.9
08	39.6	81.8	1.06	3.98	355	67	32.9	0.84	3.25	15.3	30.3	1.1	0.68	29.1	3.7	3.0
09	34.0	48.1	-	1.66	-	-	-	-	3.70	28.4	11.5	1.1	0.62	-	13.2	-
10	37.8	71.0	1.42	3.50	277	51	32.4	0.88	6.80	34.7	33.9	1.3	0.60	37.7	12.2	12.0

Table S2. Tree characteristics at Rottenburg (RO). Diameter at breast height (DBH), radial increment in 2022 estimated from dendrometer recordings (incr. 2022) and mean tree ring width of the ten years before 2022 (TR 2012-2021). For the period from April 15 to August 31, 2022: mean and maximum tree water deficit (mean TWD and max. TWD), threshold in soil water content where TWD persists (k) and coefficient of determination (R^2 [1]) for exponential between relative extractable water (REW) and TWD. Depth of the sap wood (sw depth) and relative sap wood area (swa). Mean sap flow (sf), slope of linear regression between REW and sap flow (m), its coefficient of determination (R^2 [2]), relative sap flow at threshold k (sf at k), sap flow at maximum drought (sf at max dr.) and sap flow at maximum TWD (sf at max TWD).

RO	tree height [m]	DBH [cm]	incr. 2022 [mm]	TR 2012–2021 [mm]	max. TWD [μm]	mean TWD [μm]	k [%]	R^2 [1]	sw depth [cm]	swa [%]	mean sf [Kg day^{-1}]	m [%]	R^2 [2]	sf at k [%]	sf at max dr. [%]	sf at max TWD [%]
01	30.9	46.3	3.2	4.03	745	136	23.0	0.88	6.75	49.8	67.2	1.6	0.79	53.5	22.5	19.1
02	28.9	36.6	3.2	3.21	544	78	20.3	0.90	4.60	43.9	37.5	1.2	0.58	47.4	19.1	12.6
03	25.3	37.2	2.5	3.23	309	52	21.9	0.93	5.40	49.6	29.2	1.1	0.49	52.6	22.6	6.7
04	28.3	38.3	-	2.62	-	-	-	-	4.60	42.2	36.3	1.1	0.53	-	27.5	-
05	26.9	35.7	2.0	3.38	292	60	23.4	0.86	5.55	52.6	20.8	0.6	0.59	29.0	14.1	12.2
06	28.3	43.8	2.4	3.78	635	117	20.6	0.99	4.70	38.3	45.2	1.2	0.61	43.3	13.3	14.1
07	34.8	29.8	3.0	3.11	612	171	24.2	0.93	4.25	48.9	22.7	1.4	0.78	41.6	5.1	5.0
08	28.8	38.5	3.3	5.28	704	139	21.6	0.89	4.80	43.6	40.7	1.3	0.69	53.2	20.6	18.5
09	32.4	44.6	3.2	3.88	465	86	19.5	0.90	6.65	50.8	57.4	1.0	0.45	34.7	18.1	10.4
10	27.9	37.9	3.0	4.08	530	102	21.0	0.86	6.00	51.0	1.0	0.71	40.3	17.6	12.8	

Table S3. Tree characteristics at Heinersreuth (HE). Diameter at breast height (DBH), radial increment in 2022 estimated from dendrometer recordings (incr. 2022) and mean tree ring width of the ten years before 2022 (TR 2012-2021). For the period from April 15 to August 31, 2022: mean and maximum tree water deficit (mean TWD and max. TWD), threshold in soil water content where TWD persists (k) and coefficient of determination (R^2 [1]) for exponential between relative extractable water (REW) and TWD. Depth of the sap wood (sw depth) and relative sap wood area (swa). Mean sap flow (sf), slope of linear regression between REW and sap flow (m), its coefficient of determination (R^2 [2]), relative sap flow at threshold k (sf at k), sap flow at maximum drought (sf at max dr.) and sap flow at maximum TWD (sf at max TWD).

HE	tree height [m]	DBH [cm]	incr. 2022 [mm]	TR 2012–2021 [mm]	max. TWD [µm]	mean TWD [µm]	k [%]	R^2 [1]	sw depth [cm]	swa [%]	mean sf [Kg day^{-1}]	m [%]	R^2 [2]	sf at k [%]	sf at max dr. [%]	sf at max TWD [%]
01	32.0	47.1	1.57	1.09	171	25	19.9	0.30	2.85	22.7	19.3	1.3	0.59	45.2	27.6	19.1
02	28.1	48.1	-	1.34	-	-	-	-	2.55	20.1	9.9	1.7	0.63	-	11.1	-
03	30.4	38.2	0.48	0.70	305	105	30.3	0.76	1.95	19.4	11.7	1.6	0.77	49.3	12.4	9.6
04	34.7	42.0	-	0.59	-	-	-	-	3.90	33.7	11.0	-	-	-	-	-
05	30.0	42.9	0.55	0.51	208	56	30.7	0.57	2.35	20.7	10.1	1.6	0.74	50.6	13.1	23.5
06	28.0	53.2	-	1.57	-	-	-	-	6.25	41.5	70.8	1.1	0.48	-	40.3	-
07	36.0	59.6	-	2.01	-	-	-	-	5.25	32.1	30.9	1.8	0.74	-	10.5	-
08	35.0	45.5	-	0.43	-	-	-	-	2.00	16.8	4.7	2.2	0.72	-	6.5	-
09	30.2	36.5	-	-	-	-	-	-	2.10	21.7	4.5	1.8	0.79	-	13.6	-
10	30.0	37.9	0.73	0.51	136	41	28.2	0.70	2.85	27.8	6.6	1.8	0.78	50.5	13.7	11.5

Table S4. Tree characteristics at Merzalben (ME). Diameter at breast height (DBH), radial increment in 2022 estimated from dendrometer recordings (incr. 2022) and mean tree ring width of the ten years before 2022 (TR 2012-2021). For the period from April 15 to August 31, 2022: mean and maximum tree water deficit (mean TWD and max. TWD), threshold in soil water content where TWD persists (k) and coefficient of determination (R^2 [1]) for exponential relative extractable water (REW) and TWD. Depth of the sap wood (sw depth) and relative sap wood area (swa). Mean sap flow (sf), slope of linear regression between REW and sap flow (m), its coefficient of determination (R^2 [2]), relative sap flow at threshold k (sf at k), sap flow at maximum drought (sf at max dr.) and sap flow at maximum TWD (sf at max TWD).

ME	tree height [m]	DBH [cm]	incr. 2022 [mm]	TR 2012–2021 [mm]	max. TWD [µm]	mean TWD [µm]	k [%]	R^2 [1]	sw depth [cm]	swa [%]	mean sf [Kg day^{-1}]	m [%]	R^2 [2]	sf at k [%]	sf at max dr. [%]	sf at max TWD [%]
01	33.0	41.5	-	3.53	-	-	-	-	5.05	42.7	17.1	2.1	0.89	-	11.2	-
02	32.1	36.3	-	1.83	-	-	-	-	4.25	41.4	18.2	2.3	0.84	-	11.1	-
03	32.1	46.2	-	2.77	-	-	-	-	4.80	37.2	11.2	2.1	0.93	-	5.2	-
04	32.7	47.4	-	3.89	-	-	-	-	6.75	48.8	13.0	1.9	0.88	-	8.3	-
05	33.8	52.2	-	2.93	-	-	-	-	4.50	31.5	19.9	1.6	0.89	-	2.6	-
06	33.8	52.6	-	4.07	-	-	-	-	6.65	44.2	32.6	2.2	0.89	-	5.7	-
07	33.0	46.5	3.42	3.71	272	50	26.5	0.94	5.15	39.4	15.7	2.3	0.86	50.3	4.2	4.1
08	31.0	45.5	-	4.21	-	-	-	-	6.00	45.8	14.8	0.8	0.34	-	26.6	-
09	31.7	38.3	-	1.43	-	-	-	-	3.55	33.6	3.5	2.2	0.83	-	8.6	-
10	31.5	40.5	2.87	2.91	277	58	28.4	0.95	5.35	45.9	12.2	2.5	0.88	56.7	2.6	3.1

Table S5. Alternative analysis using VSWC instead of REW for calculation of k. Average threshold in VSWC where TWD persists (k_{VSWC}), coefficient of determination (R^2) for exponential regression between TWD and VSWC, slope of linear regression between sap flow and VSWC (m) and respective coefficient of determination (R^2), mean relative sap flow at threshold k, mean relative sap flow at the 10% lowest VSWC, i.e. at maximum drought and mean relative sap flow at a relative TWD>0.9, i.e. maximum TWD. Superscript lowercase letters indicate groups that statistically differ from each other ($p<0.05$) and subscript numbers indicate the number of trees observed.

	k_{VSWC} [%]	R^2 VSWC/ TWD	m [%]	R^2 VSWC/ sap flow	sap flow at k [%]	sap flow at max drought [%]	sap flow at max TWD [%]
BB	$12.8\ _9{}^a$	$0.82\ _9$	$6.0\ _{10}{}^a$	$0.60\ _{10}$	$49.8\ _9{}^a$	$17.1\ _{10}{}^a$	$16.2\ _9{}^a$
RO	$10.6\ _9{}^b$	$0.91\ _9$	$5.1\ _{10}{}^a$	$0.63\ _{10}$	$45.3\ _9{}^a$	$16.6\ _{10}{}^a$	$13.5\ _9{}^a$
HE	$23.0\ _4{}^c$	$0.65\ _4$	$5.9\ _9{}^a$	$0.81\ _9$	$48.7\ _4{}^a$	$14.8\ _9{}^a$	$15.9\ _4{}^a$
ME	$12.4\ _2{}^a$	$0.95\ _2$	$5.9\ _{10}{}^a$	$0.75\ _{10}$	$41.7\ _2{}^a$	$8.6\ _{10}{}^a$	$3.6\ _2{}^a$
